# Community perceptions, trust, and experiences of malaria clinical research and participation in rural Cambodia

**DOI:** 10.1186/s41182-026-01069-4

**Published:** 2026-09-08

**Authors:** Bipin Adhikari, Lek Dysoley, Mom Ean, Hem Vattanak, Ung Soviet, Abhijit Mishra, Sarah Cassidy-Seyoum, Elke Wynberg, Rupam Tripura, Kamala Thriemer, Cindy S. Chu, Nicholas P. J. Day, Lorenz von Seidlein, Arjen M. Dondorp, Thomas J. Peto, Phaik Yeong Cheah

**Affiliations:** 1 Mahidol-Oxford Tropical Medicine Research Unit, Faculty of Tropical Medicine, Mahidol University, Bangkok, Thailand; 2 Centre for Tropical Medicine and Global Health, Nuffield Department of Medicine, University of Oxford, Oxford, UK; 3 CNM National Centre for Parasitology, Entomology and Malaria Control, Phnom Penh, Cambodia; 4 School of Public Health, National Institute of Public Health, Phnom Penh, Cambodia; 5 Provincial Health Department, Stung Treng, Cambodia; 6 Global and Tropical Health Division, Menzies School of Health Research, Charles Darwin University, Darwin, Australia; 7 Lao-Oxford-Mahosot Hospital-Wellcome Trust Research Unit, Vientiane, Lao PDR

**Keywords:** Malaria, Treatment-seeking behaviour, Community engagement, Clinical research, Therapeutic misconception, Cambodia

## Abstract

**Introduction:**

Understanding community perspectives on malaria treatment and research participation is important for designing ethically appropriate community engagement strategies, strengthening informed consent, and improving the conduct of clinical research while supporting disease control. This study explores treatment-seeking behaviours, perceptions of malaria, and experiences of research participation among community members in the catchment area of a rural research clinic run by the Mahidol-Oxford Tropical Medicine Research Unit (MORU) in Siem Pang district, Cambodia.

**Methods:**

This qualitative study was conducted between July and September 2025. We conducted 15 semi-structured interviews and five focus group discussions (FGDs) with 40 purposively selected participants using maximum diversity sampling, including 19 participants previously enrolled in malaria clinical trials and 21 community members familiar with MORU’s research clinic within the Siem Pang Health Centre compound. All interviews and FGDs were conducted in Khmer, the national language of Cambodia. Interviews were audio-recorded, translated into English, transcribed verbatim, and analysed thematically. Transcripts underwent thematic analysis exploring knowledge of malaria, pathways to treatment, perceptions about research staff, and experiences of research participation.

**Results:**

Respondents reported heterogeneous understandings of malaria and its transmission, which were reflected in their explanatory models and accounts of treatment-seeking behaviour. We found that treatment choices were influenced by accessibility, cost, and perceived quality. Respondents perceived MORU’s research clinic as a specialized malaria treatment centre. Some described clinical trials as routine treatment services. Respondents reported staff as caring, cautious and thorough in treatment and follow-up. Participants generally described the research activities in the Siem Pang research clinic as trustworthy, effective, and compassionate. Participants reported that research participation provided improved access to care, and close follow-up, and viewed it as contributing to malaria elimination. Participants expressed strong support for the researchers’ continued presence and expansion into broader health research and education.

**Conclusions:**

Participants perceived the research unit as providing high-quality clinical care, reflecting their experiences of sustained interactions with the research clinic and its procedures. Participants’ narratives suggested features consistent with therapeutic misconceptions and may have promoted respondents’ liberal trust and participation. Researchers should be aware of the unintended consequences of ethical research practices in remote communities. Acknowledging misplaced trust could enable wider discussion for managing community members’ perceptions of clinical trials.

**Supplementary Information:**

The online version contains supplementary material available at https://doi.org/10.1186/s41182-026-01069-4.

## Background

Over the past few decades, an increasing proportion of infectious disease research has been conducted in low- and middle-income countries (LMICs), where disease burden is high and research capacity has expanded [1–4]. While this has increased opportunities to generate locally relevant evidence, clinical research in LMICs also faces social, cultural, political, and infrastructural challenges that influence community engagement and trial implementation [2, 4–6].

Malaria, long endemic in Cambodia, is now largely confined to geographically remote regions of the country [7, 8]. Malaria incidence in Cambodia has declined substantially over the past two decades, with only 118 total malaria cases reported nationwide between January and July 2026, of which half were imported from travellers [9]. Historically, Cambodia was the epicentre of antimalarial drug resistance and its subsequent spread across Southeast Asia and westward to sub-Saharan Africa, driven by factors including prolonged antimalarial drug pressure, widespread availability of antimalarials, suboptimal treatment practices, and intense cross-border population movement [10, 11]. Considering this historical burden, Cambodia’s achievement in progressing toward elimination represents a remarkable public health milestone [7]. Historical efforts in programmatic implementation (for instance outreach programme to tackle malaria among forest goers) and malaria research have been critical cornerstones of this success, and such continued efforts remain vital to achieve elimination and subsequently prevent reintroduction [8, 12].

In collaboration with the National Centre for Parasitology, Entomology and Malaria Control (CNM), the Mahidol–Oxford Tropical Medicine Research Unit (MORU) established a small hospital-based clinical trial centre in Pailin, Cambodia, in 2007 to study artemisinin resistance [13, 14]. Since then, MORU’s research has focused on tracking antimalarial resistance and finding optimal antimalarial therapies [15]. Recent research has expanded to other areas of Cambodia and explored novel treatment options for vivax malaria [16]. Nonetheless, MORU is one among several other research organizations who partner with CNM and other stakeholders conducting a wide range of clinical and public health research.

In 2017, MORU established a research clinic in Siem Pang hosted within the premise of the district’s health centre. The MORU research clinic is located in an annexed wooden building and has its own staff. The research clinic includes a consultation room, a laboratory, and five in-patient beds, and within this set-up, health services are exclusively provided to participants of the clinical trials. Research conducted at the clinic has included (antimalarial) chemoprophylaxis among forest goers [17], evaluation of triple artemisinin-based combination therapies [15, 18], and studies to determine the optimal tafenoquine dose and evaluate alternative radical cure regimens for *Plasmodium vivax* malaria [19]. *Plasmodium vivax* can cause recurrent (relapsing) malaria due to dormant liver-stage parasites (hypnozoites), making effective radical cure essential.

Community and patient engagement have been integral to MORU’s research and are intended to promote ethical conduct, mutual trust, and meaningful community participation in clinical research [20, 21]. For instance, in recent years, post-study engagement is increasingly being promoted. A post-study engagement was conducted with patients to disseminate results [22] from vivax trial evaluating three treatment regimens [19, 23] in Cambodia. Both hospital-based and community-based clinical trials have been accompanied by sustained engagement activities to ensure ethical conduct and local participation. In 2021, the Youth Advisory Group on Health and Research Engagement (YAGHRE) was established by MORU’s researcher together with local school and health authorities, under the supervision of CNM. YAGHRE was aimed at building the capacity of local youth in health and research literacy and promoting their meaningful involvement in research [24, 25].

The YAGHRE activities can be categorized into (1) individual-level capacity building, e.g. for computer and software literacy, audio-visual material preparation and (2) contributing to MORU’s overall engagement activities (for e.g. co-creating scripts for antimicrobial resistance or AMR-drama in Battambang and Siem Reap provinces, health promotional activities such as school-based health-related presentations and discussions) and providing feedback to MORU’s research [26, 27]. These activities have helped improve research and endowed YAGHRE members to develop deeper understanding of biomedical research, enhancing their health and research literacy [24, 25]. However, despite these sustained engagement activities, little is known about how community members and research participants perceive MORU, its research activities, and the relationship between research and healthcare in this setting.

However, to date, no formal study has examined how sustained malaria research and community engagement activities in Siem Pang are perceived by research participants and the wider community, or how these experiences shape their understanding of clinical research and their relationships with research institutions. Addressing this knowledge gap is important for informing ethically appropriate community engagement strategies and the design and implementation of future clinical research in similar settings. The primary objective of this study was to explore the perspectives of participants in MORU’s malaria studies, and other community stakeholders, regarding MORU and the malaria research it conducts in Siem Pang District, Cambodia.

## Methods

### Study design

This was a descriptive qualitative study and is reported in accordance with the Consolidated Criteria for Reporting Qualitative Research (COREQ guidelines, Supplementary File 1) [28]. A thematic analysis was used to explore the topic in depth based on the interviews and focus group discussions conducted with community members.

### Study setting and health care landscape

Siem Pang is a remote district located in the northeastern part of Cambodia, bordering Lao PDR. The district is divided by the Sekong River, which feeds into the Mekong River at the provincial capital, Stung Treng. The Sekong River is both a source of food and water for farming during the rainy season. Flooding, as well as low water levels, impact access to health facilities. Cost often prevents residents from using motorized boats.

The roads connecting the villages and to the district headquarter are unpaved, which affects transportation, especially during the rainy season. The Siem Pang Health Centre offers outpatient services, care for minor injuries, and antenatal, delivery, postnatal, and immunization services. These services are constrained by the limited number of healthcare workers and low availability of medical and surgical equipment. The cost to see a health care worker is usually 2000 KHR (~ 0.50 USD), while the health care workers’ salaries are approximately 300 USD per month. Private health services are available in the form of small clinics that provide basic outpatient services. Grocery shops also sell antibiotics and a range of other commonly used medications (e.g. paracetamol, analgesics, bandages, and first-aid supplies). However, antimalarials are available for free because of the government’s subsidization. This mix of formal and informal healthcare options collectively serves the population but is neither adequate—in terms of access and service quality—nor does it provide emergency or advanced care. For higher level care, residents must travel long distances to Stung Treng, and those with greater financial resources sometimes cross the border to Vietnam.

The primary point of contact for patients with febrile illnesses, including malaria, is the village or mobile malaria workers (VMWs/MMWs), who perform rapid diagnostic tests before providing antimalarials when patients have malaria. For the purpose of research recruitments, VMWs/MMWs were asked to refer patients to MORU’s research clinic within Siem Pang Health Centre if patients had malaria. During ongoing malaria clinical trials, patients with suspected malaria or fever were screened for eligibility, followed by recruitment and consequent clinical care.

### Clinical trials conducted at the Siem Pang health centre

This study was conducted with participants in three clinical trials at the Siem Pang research clinic from 2022 until date: (1) SEADOT; (2) EFFORT and (3) DeTACT (Table 1). Patients from the community presenting to VMWs/MMWs with a positive rapid diagnostic test for malaria were enrolled into these trials at the point of diagnosis. Eligible patients for trials were compensated for time and travel costs incurred when visiting MORU’s research clinic led by local researchers and a dedicated foreign clinician. Non-eligible malaria patients were treated at the Siem Pang health centre by healthcare staff.

**Table 1:** Comparative descriptions of three MORU-led clinical trials conducted at the Siem Pang Health Centre

Characteristics	Clinical trials		
Trial acronym	SEADOT	EFFORT	DeTACT
Full trial title	Southeast Asia Dose Optimization of Tafenoquine for vivax malaria	Effectiveness of Novel Approaches to Radical Cure with Tafenoquine and Primaquine for vivax malaria	A Trial to Compare the Efficacy, Safety and Tolerability of Combinations of 3 Anti-malarial Drugs Against Combinations of 2 Anti-malarial Drugs for falciparum malaria
ClinicalTrials.gov ID	NCT04704999 (registered 14 January 2021)	NCT04411836 (registered 2 June 2020)	NCT03923725 (registered 18 April 2019)
Study design	Multicentre, randomized clinical trial	Multicentre, open-label, randomized controlled effectiveness trial	Multicentre clinical trial
Countries/sites	Multi-country; Siem Pang site in Cambodia	Multi-country; Cambodia (3 sites including Siem Pang)	Multi-country; Cambodia (2 sites including Siem Pang)
Study population	Patients with *Plasmodium vivax* malaria	Patients with uncomplicated *Plasmodium vivax* malaria	Patients with *Plasmodium falciparum* malaria
Age groups included	Adults and children (weight-based strata)	Adults	Adults
Key inclusion criteria	Confirmed *Plasmodium vivax* infection by microscopy; eligibility for tafenoquine	Uncomplicated *Plasmodium vivax* malaria; eligible for primaquine/tafenoquine	Uncomplicated *P falciparum* cases
Intervention arms	Tafenoquine 300 mg (or 5 mg/kg) versus 450 mg (or 7.5 mg/kg) single dose	Unsupervised low dose primaquine 14 days versus High dose primaquine 7 days versus Tafenoquine single dose	Artemether–lumefantrine combined with amodiaquine versus Artesunate–piperaquine combined with Mefloquine

### Study respondents

Participants were recruited using purposive sampling, with maximum diversity sampling employed to ensure variation in participant type, gender, village, education level, and prior experience with MORU and its research. The participants included (1) community members (teachers, community members, students, and youth members) and (2) participants of the above three malaria clinical trials [29]. The sample size was guided by the principle of 'data saturation', whereby recruitment and data collection continued until no new data/themes emerged across interviews and focus group discussions. BA, ME and HV assessed saturation during data collection and continued to review it during coding and team discussions. They compared emerging information and themes across respondents and reached consensus that these were recurring and that subsequent interviews were not generating substantively new information or themes. Saturation was thus assessed across the dataset rather than separately for clinical trial participants and community members [30]. A total of 40 individuals were recruited in this study following the tenet of stakeholder analysis and maximum diversity sampling. The maximum diversity sampling approach was chosen to retain the heterogeneity of respondent type, gender, villages and education level including discussions to facilitate open discussion (Table 2) [31].

**Table 2:** Socio-demographics of participants in the study

Study respondents	Interviewee ID	Age	Gender	Education (years)	Occupation
Community members	PE-CM-01	28	M	Bachelor degree	Secondary school teacher
	PE-CM-02	27	M	Bachelor degree	Secondary school teacher
	PE-CM-03	29	M	Bachelor degree	High school teacher
	PE-CM-04	31	F	Bachelor degree	Secondary school teacher
	PE-CM-05	37	M	Nursing degree	Nurse
	PE-CM-06	40	M	Grade 2	Businessman
	PE-CM-07	30	M	Bachelor degree	Secondary school teacher
	PE-CM-08	34	M	Bachelor degree	High school teacher
	PE-CM-09	54	M	Grade 4	Commune council officer and farmer
	PE-CM-10	40	M	Grade 12	District council officer and farmer
	PE-CM-11	84	M	Grade 12	Farmer
	PE-CM-12	35	F	Grade 12	District officer
	PE-CM-13	54	M	Grade 6	Employee of NGO and farmer
	PE-CM-14 (dyad)	45	M	Grade 4	Famer
		41	F	Grade 1	Farmer
	PE-CM-15	52	M	Nurse’s degree	Siem Pang Health Centre staff
	FGD (PE-CM-16)	18	M	Grade 10	High school student
		18	M	Grade 11	High school student
		20	M	Grade 11	High school student
		18	F	Grade 10	High school student
		19	F	Grade 11	High school student
		18	F	Grade 11	High school student
Malaria research participants	FGD 01 (RP-01) DeTACT	30	F	Grade 2	Farmer
		34	M	N/A	Farmer
		34	M	Grade 2	Farmer
		23	M	Grade 12	Farmer
	FGD 02 (RP-02) EFFORT-FGD-I	26	M	Grade 12	Solider
		30	M	Grade 12	Solider
		40	M	Grade 12	Solider
		37	M	Grade 12	Solider
		29	M	Grade 12	Solider
	FGD 03 (RP-03) EFFORT-FGD-II	33	F	Grade 12	School teacher
		23	F	Grade 12	School teacher
		22	M	Grade 12	Employee of NGO
		59	M	Grade 5	Farmer
		20	M	Never attended school	Farmer
	FGD 04 (RP-04) SEADOT	40	M	Bachelor degree	Soldier
		32	M	Grade 12	Soldier
		25	M	Never attended school	Farmer
		44	F	Never attended school	Farmer
		28	F	Grade 2	Farmer

The inclusion criteria included individuals aged 16 years or older who had either participated in one of the three MORU clinical trials or were identified as key stakeholders (e.g. teachers, youth members, and other community members familiar with MORU’s research activities) because of their experience with or knowledge of MORU’s research and community engagement in Siem Pang. Potential participants were identified by the local research team (ME and HV) using MORU clinical trial records for previous trial participants and their knowledge of the local community for community members and key stakeholders. Key stakeholders were purposively selected based on their roles and experience relevant to malaria research and community engagement in Siem Pang. Potential respondents were then approached by ME or HV, informed about the study, and asked whether they were interested in participating. Those who expressed interest were invited to the MORU research clinic for the interview or FGD. Of the 50 individuals contacted, five reported that they were unable to participate, and four did not attend the scheduled interview.

Based on their categorical relevance to our research question, potential respondents were invited for participation. Among the participants, 19 had participated in one of the three malaria clinical trials. Table 2 provides the demographic details of participants’ characteristics.

After explaining the purpose of the study based on the participant information sheet, written informed consent was obtained from each participant.

### Data collection and interview guide

Semi-structured interview (SSI) and focus group discussion (FGD) guides were developed based on the study objectives, overarching themes aligned with the research question, and the published literature on malaria research [16, 32], community engagement [20, 21, 33, 34], and research ethics [33, 35] (Supplementary File 2). The guides were reviewed by the YAGHRE members and adapted to suit individual interviews and group discussions. The same literature informed both guides, which were adapted to suit individual interviews and group discussions. Semi-structured interviews were used to explore individual experiences in depth, whereas FGDs were used to elicit shared community perspectives and discussion among participants with similar backgrounds. The guide was pre-tested through initial interviews with two researchers and four community stakeholders who were not included in the final study. Feedback from the pilot interviews was used to improve the clarity and comprehensibility of the questions before data collection commenced. During data collection, only the wording of prompts and follow-up questions was refined iteratively, while the same core themes and questions were retained throughout all interviews and FGDs.

Between July and September 2025, we conducted 15 semi-structured interviews with community members and five FGDs. One FGD included six community members (school students), while the four FGDs with previous clinical trial participants comprised four (DeTACT), five (EFFORT group-I), five (EFFORT group-II), and five (SEADOT) participants, respectively. Participants were grouped according to their recruitment category and, for clinical trial participants, by the trial in which they had previously participated. The interviews and FGDs lasted between 45 and 120 min. Interviews were conducted in Khmer, the national and widely spoken language, by ME and HV at the MORU’s clinic. In one FGD, real-time translation into English was provided for BA. To minimise loss of meaning, ME and HV subsequently reviewed the translated transcripts against the original Khmer audio recordings during transcription and translation. ME is a former female midwife who has worked with various development organizations and is familiar with Siem Pang, which is also her hometown. HV is a fellow male Cambodian who has worked as a research assistant since the establishment of MORU in Siem Pang and is well recognised for good rapport and approachability. BA is a Nepali clinician specialising in Tropical Medicine and Social Sciences, with more than a decade of experience in qualitative research methods. ME and HV were employed by MORU and involved in its community engagement and research activities in Siem Pang, whereas BA was affiliated with MORU as the study’s principal investigator. All audio-files were translated and transcribed verbatim into English for analysis. A notetaker from within the team also recorded key takeaways arising during the interviews. All transcripts and the interviewer’s notes were first cross-checked with the audio-recordings.

### Data analysis

Transcripts and the notes were read thoroughly and coded line by line using the qualitative data analysis software NVivo 12 (QSR International, Doncaster, Australia). The initial codebook based on the interview guide (deductive approach) was revised based on the emerging themes from the data (inductive approach). Transcripts and notes were coded in NVivo 12 by BA and were reviewed by EM and HV. The coding framework, coding patterns, and supporting quotations were reviewed by EM and HV against the original transcripts, and any discrepancies were resolved through discussion and consensus. Data analysis followed Braun and Clarke’s thematic analysis process, reiteratively refining the codes and themes relevant for the research question [36]. Regular debriefings were held between the team members concerning the final themes and their interpretation to reach a consensus. To address the research questions, themes and the supporting quotes were presented as the findings of this study.

## Results

### Overview

Our findings revealed a spectrum of themes related to health literacy, pragmatic decision-making in care-seeking, and perceptions on MORU’s quality of service and ethics of care. Themes included (1) knowledge and perceptions of malaria; (2) treatment-seeking pathways and experiences; (3) impressions of the research centre and its services, (4) understanding and reflections on research participation and (5) follow-up and perceived efficacy of treatment; and (6) recommendations for future engagement.

### Understanding and perceptions of malaria

Participants demonstrated heterogeneous understandings of malaria transmission and prevention. While many linked malaria to mosquito bites and forest exposure, others expressed misconceptions, such as associating malaria transmission with drinking contaminated water. There was an understanding that mosquito eggs could be found in water which may have been derived from the widespread public health messages related to dengue in the community. Almost all participants were aware that malaria was associated with spending time in the forest, and many community members did visit the forest seasonally for food and collection of special fruits and timber.

*“It might be transmitted by mosquitoes or drinking unclean water which has mosquitoes’ eggs in it.” (Malaria Research Participant–DeTACT)*

These mixed perceptions reflect both lived experience and local explanatory models of disease. Despite this variation, participants consistently recognized malaria as a recurrent and burdensome illness. When asked if and how many times, they had malaria, one participant said:

*“Countless, since I was 12 years old, I started having this disease, shaking and likelihood to have a seizure.” (Malaria Research Participant–DeTACT)*

Some had numerous malaria infections, and often were the frequent forest goers and lived near the forest fringe while others did not. These repeated malaria episodes and treatment were a concern for participants, particularly when recurrent episodes required them to make repeated visits for care. Such accounts of suffering from malaria also highlight how malaria is embedded in their living shaping participants’ interpretations of illness and their engagement with care services.

### Treatment-seeking pathways and experiences

Respondents reported diverse treatment pathways shaped by accessibility (road conditions, means of transport), resources, and perceived quality of care. Health centres were often the first point of contact for the formal health services, but logistical barriers such as transport and cost influenced decisions to seek treatment elsewhere (for e.g. private clinics, and higher health services).

*R2: “I didn't go to the hospital. I didn't have a motorbike because my child took it to go to school, so I just bought medicine at the clinic.” (Malaria Research Participant–DeTACT)*

While many sought care from village malaria workers (VMWs) or military health posts, they perceived that MORU’s research clinic in Siem Pang was offering specialized and high-quality treatment, including for recurrent cases of malaria such as when suffering from vivax malaria. Experiences of repeated malaria episodes often informed a sense of vulnerability and resignation, yet the accessibility of the Siem Pang clinic provided reassurance and trust in the health system.

While the majority of patients went to VMWs as first point of contact, some participants were military personnel, and initially sought care at their own clinic within the barracks. Only after referral did they come to the MORU research clinic. Some participants described how they self-treated malaria at home, often following recommendations from friends and families before seeking care from VMWs or the health centre.

*“When I came back from the forest, I said I’m a little bit not well, I thought it was a normal cold. Me and other 6 people when we came from the forest, they [community members] asked me to drink about one case of beer, and I was still shaking.” (Malaria Research Participant–DeTACT)*

These recursive treatment pathways often involved a common trajectory of self-diagnosis and self-treatment preceding professional care, highlighting how treatment-seeking behaviour is shaped by social and community practices.

### Impressions of MORU’s research clinic and its role in malaria care

While some participants could delineate health care versus clinical research in Siem Pang, some thought better health services were provided by the MORU research facility due to its specialization in malaria treatment. Initially, some viewed MORU as a “clinic” or “treatment agency”. Engagement with its study staff transformed their understanding of how research and health promotion were conducted through youth engagement initiatives.

*“Against the initial impression of MORU as an embedded clinic or health service institution, their outreach to remote communities where YAGHRE members presented and spread the knowledge on diseases were particularly appreciated by the parents as well.” (PE-CM-10)*

Care provided during research was for most participants positively perceived in contrast to usual care at the health centre where they would often have to face queues, appointment tickets, and navigation through testing before finally obtaining services. At the research clinic, participants often felt less rushed, some experienced staff’s demeanours as comforting, while others anticipated the cure for their illnesses.

*“I: And how did you feel when you first stepped into the MORU?*

*R1: It was convenient.*

*I: How did you feel?*

*R2: I felt comfortable all of a sudden.*

*I: How did you feel? All of you?*

*R4: I felt like when I came to MORU, my symptoms will be recovered.” (Malaria Research Participant–DeTACT)*

In addition, going through the patient information sheet and requiring consent before medical services gave participants the impression of attentiveness and clear communication. Such encounters and subsequent follow-up (all part of research) made the patients feel cared for.

*“He explained to us when we were confused, like we didn't remember where he told us how to take the medicine, so he told us again. If we already took the medicine and came to follow up, he asked us if we were feeling well or not. If we weren’t feeling better, he will check our symptoms again.” (Malaria Research Participant–DeTACT)*

Participants spontaneously compared the research clinic with the routine health care they had received in the past. One of the prominent distinctions patients made was around how repeated cases of malaria were cured after receiving treatment from the research clinic. Some other participants expanded their views on how the research clinic was reaching out to the community through school engagement activities.

*“For me, I choose MORU because MORU has a method for treating patients to be completely cured. So, I'm happy to be treated at MORU.” (Research Participant–EFFORT)*

*“MORU is an agent for treating malaria and for educating people about malaria in each community. I saw them go to schools in the community, and there was a youth volunteer group of the MORU organization in remote communities.” (Malaria Research Participant–EFFORT)*

These reflections suggest that MORU research clinic’s integrated approach, comprising treatment, follow-up, and community engagement as a part of clinical trials, may have enhanced both perceived and actual quality of care in remote regions.

MORU’s field outreach, including deploying mobile malaria services to hotspots and border areas, was especially appreciated by military personnel and remote communities. Beyond reaching community members, amidst the concentrated cases of malaria among military personnel, the research clinic intensified its efforts to recruit military personnel into the study. Nonetheless, abiding to the military policies, research staff quickly relaxed its conventional methods of recruitment, treatment and follow-up at the research clinic. By being flexible in the study procedures, offering access to treatment “at the doorstep”, which was viewed as both convenient and lifesaving.

*“For my unit before, there were a lot of malaria cases and after a lot of treatment with MORU, everyone was completely cured and he thought it was good.” (Malaria Research Participant–EFFORT)*

Such accounts of research recruitment when combined with perceived care and compassion may have promoted a unique impression of the MORU’s research clinic, among both the community members and the military personnel.

### Understanding and experiences of research participation

Although not all participants could recall the procedural details of their research participation, they generally expressed satisfaction with the care and follow-up they received. Many distinguished the research clinic’s attentive, free, and consistent service from routine clinical encounters.

*“Because the service is convenient, [MORU] don't charge money from us and [we] don’t pay for the service fee. Their work is clear and they do it right.” (Malaria Research Participant–DeTACT)*

Some participants vividly remembered their initial apprehension upon entering the research process because of their unfamiliarity with the research clinic and staff. Some described their apprehension improving at the clinicians’ good bedside manner.

*“I was nervous too, to be honest. I was afraid of injections. Just seeing the needle made me nervous. I also thought I might have to sleep alone, and that made me feel scared. But the doctor was really friendly and took care of us. They gave us towels and even wiped us. If we needed something, he responded right away. All the staff were very kind and attentive.” (Malaria Research Participant–SEADOT)*

A recurring theme was the perception of MORU’s research as both a health service and an altruistic effort to help poor or rural communities. Some of these participants linked research clinic’s efforts to the higher aims of the national malaria control programme to eliminate malaria in Cambodia. These aims may have been retained by the community members, likely being articulated during engagement activities since 2018—which include the village drama against malaria (2018) in the community and study-specific activities till date [21].

*“The MORU organization also wants to eliminate malaria and wants to help the poor people who don't have money when they get sick and don't have money to go to the hospital. The MORU organization will help the poor people so that people can get free treatment and they also provide other support.” (Malaria Research Participant–EFFORT)*

*“I am not interested; I only know that MORU has a purpose of treating diseases and aims to cure people and we want to be treated to be completely cured. And the second is the desire to eliminate malaria.” (Malaria Research Participant–EFFORT)*

### Follow-up and perceived efficacy of treatment

While some participants interpreted follow-up visits as part of the research/study, others conflated the caring aspects embedded within such procedures as higher quality of care. One such participant, who was in the EFFORT study, vividly recalled the follow-up schedule and was even able to express the study purpose, in their own terms.

*“For me, I know because the medicine has 14 days, 7 days, 3 days, they want to know the stages of the medicine, they want to know the effectiveness of the medicine and our health, whether to take 7 days or 3 days, which one is better.” (Malaria Research Participant–EFFORT)*

The follow-up process was widely interpreted as a sign of care and thoroughness rather than a procedure of the study. Participants valued the regular contact as an indication of professional (clinical) commitment and as a mechanism for ensuring complete recovery.

*“R1:… for treatment, they just give us medicine and don’t follow up. They don’t know it get better or not and it can relapse. But for study, the doctor follows up long time and want to know the effective of the medicine.*

*I: and was it difficult?*

*R2: I don't think it is difficult because I always come when he made appointment with me, and because I wanted to know if I completely cured or not and was there [whether there was] something wrong with my health.” (Malaria Research Participant–EFFORT)*

Participants often differentiated study drugs from those received elsewhere, associating them with greater efficacy and fewer side effects.

*“I think the medicine at MORU is different from the medicine at the health centre and the private clinic because the medicine at MORU doesn't hurt when I take it. I take it normally and it's cured me… For the medicine at the health centre, I've seen villagers sometimes they go to the health centre or to a private clinic to get the medicine. Sometimes a week later, they had malaria again… However, for the medicine at MORU, I haven't had malaria again since I took it.” (Malaria Research Participant–EFFORT)*

Research participants’ perceptions show that MORU’s role in Siem Pang was not viewed as an isolated research organization but more as a dedicated health service provider.

### Recommendations for future engagement

Participants expressed strong support for the continuation and expansion of research activities in Siem Pang district. Their recommendations centred on sustaining YAGHRE’s impact, broadening youth participation, enhancing local health education, and extending Siem Pang clinic’s research presence to address emerging health priorities.

Some health care workers and community members, aware of the work on malaria, also advocated for the continuation of broader health and research work in Siem Pang, underscoring the scarcity of comparable health services in the area. They saw ongoing research as vital to improving community health and expanding understanding of diseases beyond malaria.

*“Even though the malaria work is over, there are other jobs…..……… Not only does it [MORU] help with malaria, there are infectious diseases, non-infectious diseases… Please continue to educate even [though] the malaria disease is over.” (PE-CM-16)*

Participants also called for new studies and continuation of free, high-quality treatment, linking research with public benefit. Some echoed the national goal of malaria elimination while encouraging a broader scope of work to other infectious diseases that are still a burden to these remote populations.

*“I would like to request that the project should continue to research new diseases and share that knowledge widely. And my goal is for the whole world to be malaria-free by the end of 2025.” (Malaria Research Participant–EFFORT)*

## Discussion

This study found that community members and previous malaria trial participants generally viewed MORU as a trusted provider of malaria care and research. Trust was shaped by positive experiences of treatment, follow-up, and community engagement, but for some participants this also blurred the distinction between clinical care and research participation. Community members’ experiences with the Siem Pang Health Centre and MORU’s research clinic highlight the impression developed progressively over the years because of their interaction with the research clinics and staff. The integrated approach, linking clinical research, community outreach, and youth engagement, may have enhanced institutional credibility and improved health and research literacy, while simultaneously blurring the distinction between clinical care and research participation [24]. The research clinic in Siem Pang is set to end by the end of 2026; nonetheless, these findings emphasize the role of and need for sustained community engagement and capacity building for ethical global health research [33, 34].

### Trust and therapeutic misconception

**Fig. 1:**
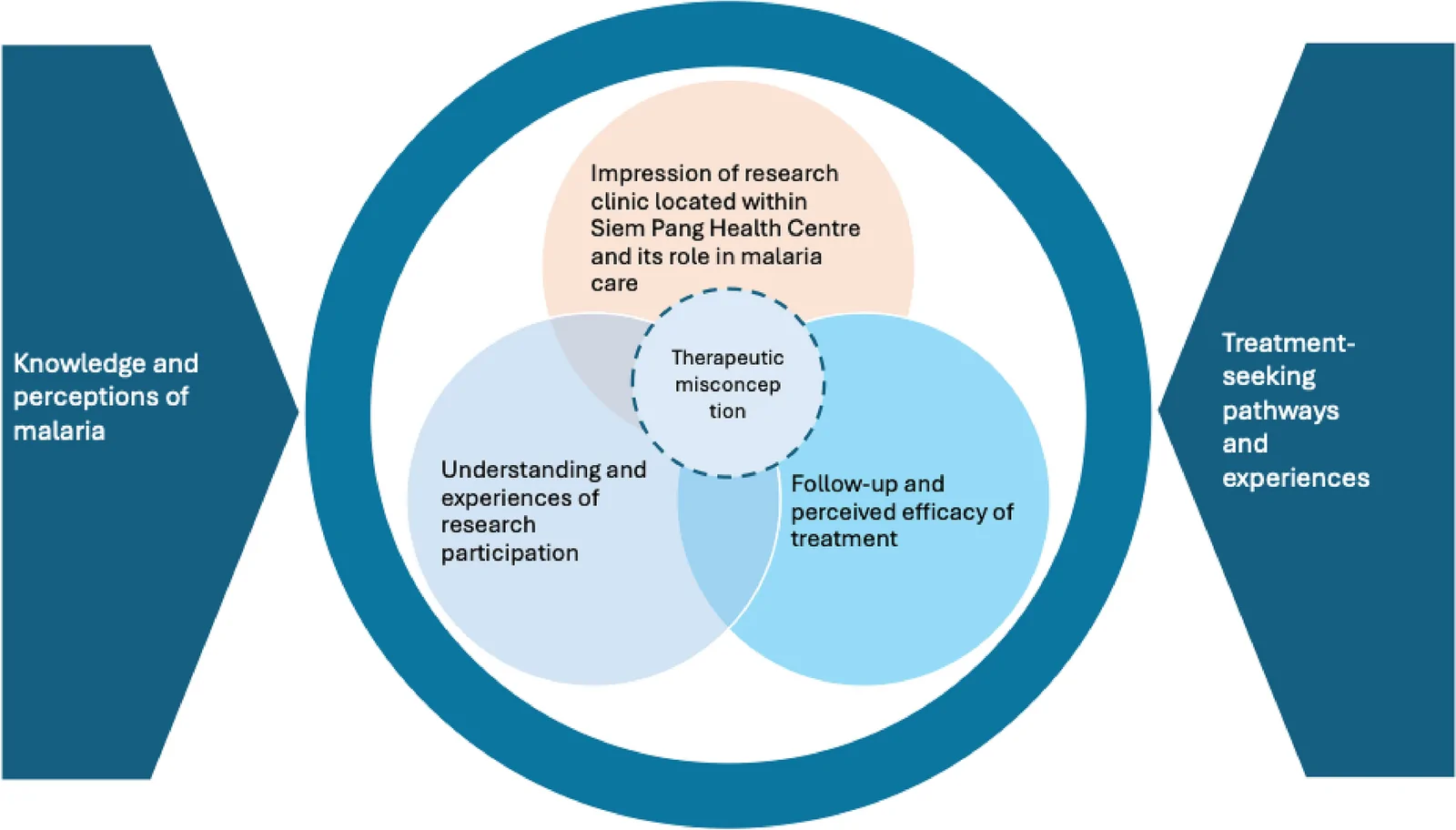
Genesis of therapeutic misconception in malaria trials conducted in research clinic within Siem Pang Health Centre

Participants expressed trust in the research clinic and its research activities. This trust was grounded in the conflation of perceived clinical care with research procedures. As much as researchers may desire complete understanding by participants regarding research procedures (through written informed consent process), clinical trials cannot occur in a silo or an isolated environment. The augmented attention to patient care in the research setting is often driven by the research procedures (e.g. informed consent, study monitoring, requiring follow-up), and these occur in controlled environments (space, time and enrolment volumes) sometimes leading to what ethicists describe as “therapeutic misconception” [37, 38]. Therapeutic misconception is a research participant's mistaken belief that a clinical trial or experimental study is primarily designed to provide them with individualized medical treatment [39]. Participants' narratives suggest features consistent with therapeutic misconception, which may have been influenced by (1) the impression community members had about research clinic embedded within the Siem Pang Health Centre; (2) their own understanding and experience of research and participation; and (3) follow-up procedures of the trials (Fig. 1). More broadly, these factors were likely shaped by local knowledge and perceptions of malaria, as well as treatment-seeking behaviours [39, 40].

When research participants have difficulty distinguishing between clinical trials and routine medical treatment, it can compromise the validity of written informed consent, failing to conceive the aim of the study [39]. Such misconceptions are influenced by contextual factors, including setting, time, and the quality of interactions, particularly when research is conducted within healthcare institutions and led by healthcare providers. Their widespread and context-dependent occurrence cautions against oversimplified interpretations of their presence. In this study, many participants demonstrated awareness that they were participating in research, yet some narratives also suggested overlap between perceptions of research participation and clinical care.

International research organizations in low- and middle-income countries are often perceived locally as providers of high-quality health services rather than pure research organizations. Prominent examples include Kenya Medical Research Institute (KEMRI) [35, 41] and Medical Research Council (MRC) UK in the Gambia [42, 43]. Health care workers and health care institutions, by virtue of their services, tend to promote both individual and institutional goodwill and trust [44–46].

The sense of reassurance that participants described when engaging with MORU suggests that institutional trust can emerge from consistency, in our case presence over the long period of time, transparency, and demonstrated commitment to participants’ well-being [44]. Similar patterns have been observed in qualitative studies from other malaria-endemic countries, where enduring relationships between researchers and communities form the foundation for ethical and sustainable research [47]. Nonetheless, a few respondents expressed concern about MORU’s declining research and engagement activities in Siem Pang as malaria incidence decreased. Fluctuations in funding, research, and engagement risk creating siloed, short-term initiatives that are difficult to sustain beyond project timelines, potentially undermining institutional reliability and long-term trust in research efforts. The shrinking funding for research also aligns with the current landscape of overall global health research.

In this context, trust extends beyond individual interactions to build institutional identity, echoing the concept of trust ‘emerging’ and ‘reciprocating’, that is, individual trust affecting institutional trust and vice versa [48, 49]. Participants viewed MORU as health care providers, research partners, and collaborators with the Centre for Malaria and Parasitology (CNM’s) of malaria elimination goals [7]. This alignment and co-partnership between local and institutional objectives also likely reinforced participants’ confidence in the research process and in MORU’s intentions.

### Understanding of research and participation motivations

Respondents’ heterogeneity in understanding of clinical research may correlate with education, exposure, and prior engagement. While some associated participation primarily with treatment access and recovery, others demonstrated awareness of research objectives—such as comparing drug regimens or evaluating medicine effectiveness. Heterogeneity in the understanding of research and conflating the objectives of research with therapeutic care are common and demand more attention from researchers [33, 41, 50]. Understanding how research procedures may be perceived as therapeutic care can help researchers identify participants’ vulnerabilities and mitigate the risks of exploitation and other ethical concerns [33, 51]. Many respondents perceived their involvement as contributing to the collective effort to eliminate malaria. Such altruistic motivations are often contingent on self-interest (perceived benefit or perceived lack of harm), highlighting a need to explore participants’ perceptions and their co-existence (of benefit and lack of harms) [52, 53].

Research participation in resource-limited settings can carry both practical and social meanings [33, 54, 55]. Even when participants did not fully remember all research procedures, their reflections indicated an understanding of broader social purpose and shared benefit. This resonates with global qualitative literature on malaria trials, which suggests that local conceptualizations of research often integrate care, reciprocity, and communal responsibility rather than being limited to scientific abstraction [56, 57].

While some overlap between research and treatment experiences was evident, respondents largely framed their study participation in terms of trust, sense of gratitude, and a desire to help others [32, 54, 58]. This relational framing underscores the importance of ongoing dialogue to ensure that informed consent and participant comprehension are both contextually grounded (warranting relational dimension) and ethically meaningful [42, 59, 60]. At a fundamental level, researchers’ awareness of these issues can help prepare local staff and strengthen safeguarding measures to ensure the ethical conduct of research whilst managing misplaced trust.

### Implications for research practice

These findings highlight the importance of ongoing communication throughout the research process, rather than relying solely on informed consent at enrolment. Reinforcing the distinction between research and clinical care during follow-up and community engagement activities may help minimise therapeutic misconception while maintaining community trust. First, community perceptions of research clinics appear to be shaped primarily by trust in the institution, reinforced through interactions with research staff. Community engagement should therefore remain central to building and sustaining trust, while also enhancing awareness among researchers and local staff of potential misinterpretations of research activities. Even when such interpretations are rooted in trust and longstanding relationships, research teams can address them through socially and culturally appropriate approaches that reduce the risk of exploitation and promote ethically responsible stewardship of participants’ trust. Second, communication strategies should address both biomedical and social dimensions of understanding, recognizing that communities interpret research through social and relational frameworks. Awareness of these ethical concerns may help researchers anticipate and mitigate potential vulnerabilities among participants. Future research could examine how community engagement models like YAGHRE influence perceptions of research over time and how institutional trust interacts with declining malaria epidemiology and associated research.

### Limitations

This study has several limitations. The investigators who recruited, interviewed, coded, and interpreted the data are affiliated with the MORU research clinic that the study evaluates, and one of the focus groups had themselves received free care from that clinic. This creates a substantial risk of social desirability and courtesy bias; respondents may have reported more favourable views to an affiliated team [61, 62], which is especially salient because therapeutic misconception is a central finding [37, 63]. The research environment and the position of researchers thus may have generated researchers’ positionality bias [64]. Acknowledging this bias, we actively reflected on positionality through reflexive practice [65] and sought to mitigate its influence by conducting interviews in Khmer through research staff who were not PIs. Independent coding by BA, followed by review and discussion with ME and HV, may have provided an additional opportunity to identify and address potential individual researcher bias. In addition, including community members who had not participated in the trial provided perspectives independent of direct trial participation, potentially offering a more balanced understanding of perceptions of the research and trust in MORU. These steps do not remove the shared institutional perspective of the research team. The findings therefore represent the perspectives respondents chose to share with a MORU-affiliated team, and the strongly positive accounts in particular should be interpreted with this caveat. A further limitation is that interviews conducted in Khmer were analysed in English transcriptions, which may have incurred translation bias missing the nuances in subsequent interpretations [66].

## Conclusions

Participants’ narratives underscore that trust, engagement, and relationships were central to the execution of clinical trials related to malaria conducted in Cambodia. Trust in the research clinic was shaped by both community engagement and positive experiences of care, although distinctions between research and routine healthcare provision were sometimes blurred. Sustained community engagement, researcher awareness of the concepts of therapeutic misconception, and transparent communication can guide ethical research practices that steward community trust.

## Supplementary Information

Supplementary Material 1. COREQ: COnsolidated criteria for REporting Qualitative research

Supplementary Material 2. FGD and SSI interview guide for the study

Supplementary Material 3. Thematic landscape, codebook, and memos of the study

## Data Availability

Because of the nature of the qualitative data in this study, even if the data are anonymized, potential respondents are identifiable. Both MORU and local ethics committee restrict the sharing of data that can potentially identify the respondents. The data are available upon request to the Mahidol Oxford Tropical Medicine Research Unit Data Access Committee (datasharing@tropmedres.ac) complying with the data access policy on case-by-case analysis (https://www.tropmedres.ac/units/moru-bangkok/bioethics-engagement/data-sharing/moru-tropical-network-policy-on-sharing-data-and-other-outputs).

## Abbreviations

AMR: Antimicrobial resistance
CNM: National Centre for Parasitology, Entomology and Malaria Control
COREQ: Consolidated criteria for reporting qualitative research
DeTACT: A Trial to Compare the Efficacy, Safety and Tolerability of Combinations of 3 Anti-malarial Drugs Against Combinations of 2 Anti-malarial Drugs for *P. falciparum* Malaria
EFFORT: Effectiveness of Novel Approaches to Radical Cure with Tafenoquine and Primaquine for *P. vivax* Malaria
FGD: Focus group discussion
LMICs: Low- and middle-income countries
MMW: Mobile malaria workers
MORU: Mahidol-Oxford Tropical Medicine Research Unit
NECHR: National Ethics Committee for Health Research (Cambodia)
OxTREC: Oxford Tropical Medicine Research Ethics Committee
SEADOT: Southeast Asia Dose Optimization of Tafenoquine for Vivax Malaria
UNESCO: United Nations Education, Scientific and Cultural Organization
VMW: Village Malaria worker
YAGHRE: Youth Advisory Group on Health and Research Engagement
