# Supplementary material for: Community perceptions, trust, and experiences of malaria clinical research and participation in rural Cambodia
Source: Trop Med Health. 2026 Sep 8;54:193. doi: 10.1186/s41182-026-01069-4 (PMC13551720; doi:10.1186/s41182-026-01069-4)
Supplement: Supplementary file 1 [file 41182_2026_1069_MOESM1_ESM.docx]

# How is malaria research perceived in Cambodia? a qualitative study among study participants and community members

Bipin Adhikari^1,2†^, Lek Dysoley^3,4†^, Mom Ean^1†^, Hem Vattanak^1^, Ung Soviet^5^, Abhijit Mishra^1^, Sarah Cassidy-Seyoum^6^, Elke Wynberg^1^, Rupam Tripura^1,2^, Kamala Thriemer^6^, Cindy S Chu^2,7^, Nicholas PJ Day^1,2^, Lorenz von Seidlein^1,2^, Arjen M Dondorp^1,2^, Thomas J Peto^1,2^, Phaik Yeong Cheah^1,2^

^1^Mahidol-Oxford Tropical Medicine Research Unit, Faculty of Tropical Medicine, Mahidol University, Bangkok, Thailand

^2^Centre for Tropical Medicine and Global Health, Nuffield Department of Medicine, University of Oxford, Oxford, UK

^3^CNM National Centre for Parasitology, Entomology and Malaria Control, Phnom Penh, Cambodia

^4^School of Public Health, National Institute of Public Health, Phnom Penh, Cambodia.

^5^Provincial Health Department, Stung Treng, Cambodia

^6^Global and Tropical Health Division, Menzies School of Health Research, Charles Darwin University, Darwin, Australia,

^7^Lao-Oxford-Mahosot Hospital-Wellcome Trust Research Unit, Vientiane, Lao PDR

†Equal contributors

[*Bipin@tropmedres.ac](mailto:*Bipin@tropmedres.ac)

**Journal**: Tropical Medicine and Health

| **Section/Topic** | **Item No** | **Checklist item** | **Reported on page No** |
| --- | --- | --- | --- |
| Domain 1: Research team and reﬂexivity | | | |
| Personal Characteristics | | | |
| Interviewer/facilitator | 1 | Which author/s conducted the interview or focus group?Interviewer/facilitator | 9 |
| Credentials | 2 | What were the researcher’s credentials? E.g. PhD, MD | 9 |
| Occupation | 3 | What was their occupation at the time of the study? | 9 |
| Gender | 4 | Was the researcher male or female? | 9 |
| Experience and training | 5 | What experience or training did the researcher have? Relationship with participants | 9 |
| Relationship with participants | | | |
| Relationship established | 6 | Was a relationship established prior to study commencement?  *Since some were trial participants and others were locals from this small town of Siem Pang, so they do know the interviewers in various extent.* | 8 |
| Participant knowledge of the interviewer | 7 | What did the participants know about the researcher? e.g. personal goals, reasons for doing the research | 8 |
| Interviewer characteristics | 8 | What characteristics were reported about the interviewer/facilitator? e.g. Bias, assumptions, reasons and interests in the research topic | 8 and 9 |
| Domain 2: study design | | | |
| Theoretical framework | | | |
| Methodological orientation and  Theory | 9 | What methodological orientation was stated to underpin the study? e.g. grounded theory, discourse analysis, ethnography, phenomenology, content analysis | 5 and 6 |
| Participant selection | | | |
| Sampling | 10 | How were participants selected? e.g. purposive, convenience, consecutive, snowball | 8 |
| Method of approach | 11 | How were participants approached? e.g. face-to-face, telephone, mail, email | 8 |
| Sample size | 12 | How many participants were in the study? | 8 |
| Non-participation | 13 | How many people refused to participate or dropped out? Reasons? | 8 |
| Setting of data collection | 14 | Where was the data collected? e.g. home, clinic, workplace | 9 |
| Presence of non-participants | 15 | Was anyone else present besides the participants and researchers? | 9 |
| Description of sample | 16 | What are the important characteristics of the sample? e.g. demographic data, date | 8 and Table 2 |
| Data collection | | | |
| Interview guide | 17 | Were questions, prompts, guides provided by the authors? Was it pilot tested? | 9 |
| Repeat interviews | 18 | Were repeat interviews carried out? If yes, how many? | NA |
| Audio/visual recording | 19 | Did the research use audio or visual recording to collect the data? | 9 |
| Field notes | 20 | Were ﬁeld notes made during and/or after the interview or focus group? | 9 |
| Duration | 21 | What was the duration of the interviews or focus group? | 9 |
| Data saturation | 22 | Was data saturation discussed? | 8 |
| Transcripts returned | 23 | Were transcripts returned to participants for comment and/or correction? | NA |
| Domain 3: analysis and ﬁndings  Data analysis | | | |
| Number of data coders | 24 | How many data coders coded the data? | 10 |
| Description of the coding tree | 25 | Did authors provide a description of the coding tree? | 10 and S3 |
| Derivation of themes | 26 | Were themes identiﬁed in advance or derived from the data? | 10 |
| Software | 27 | What software, if applicable, was used to manage the data? | 10 |
| Participant checking | 28 | Did participants provide feedback on the ﬁndings? | NA |
| Reporting | | | |
| Quotations presented | 29 | Were participant quotations presented to illustrate the themes / ﬁndings? Was each quotation identiﬁed? e.g. participant number | 10-15 |
| Data and ﬁndings consistent | 30 | Was there consistency between the data presented and the ﬁndings? | 10-15 |
| Clarity of major themes | 31 | Were major themes clearly presented in the ﬁndings? | 10-15 |
| Clarity of minor themes | 32 | Is there a description of diverse cases or discussion of minor themes? | 10-15 |
