# Supplementary material for: Community perceptions, trust, and experiences of malaria clinical research and participation in rural Cambodia
Source: Trop Med Health. 2026 Sep 8;54:193. doi: 10.1186/s41182-026-01069-4 (PMC13551720; doi:10.1186/s41182-026-01069-4)
Supplement: Supplementary file 2 [file 41182_2026_1069_MOESM2_ESM.docx]

# How is malaria research perceived in Cambodia? a qualitative study among study participants and community members

Bipin Adhikari^1,2†^, Lek Dysoley^3,4†^, Mom Ean^1†^, Hem Vattanak^1^, Ung Soviet^5^, Abhijit Mishra^1^, Sarah Cassidy-Seyoum^6^, Elke Wynberg^1^, Rupam Tripura^1,2^, Kamala Thriemer^6^, Cindy S Chu^2,7^, Nicholas PJ Day^1,2^, Lorenz von Seidlein^1,2^, Arjen M Dondorp^1,2^, Thomas J Peto^1,2^, Phaik Yeong Cheah^1,2^

^1^Mahidol-Oxford Tropical Medicine Research Unit, Faculty of Tropical Medicine, Mahidol University, Bangkok, Thailand

^2^Centre for Tropical Medicine and Global Health, Nuffield Department of Medicine, University of Oxford, Oxford, UK

^3^CNM National Centre for Parasitology, Entomology and Malaria Control, Phnom Penh, Cambodia

^4^School of Public Health, National Institute of Public Health, Phnom Penh, Cambodia.

^5^Provincial Health Department, Stung Treng, Cambodia

^6^Global and Tropical Health Division, Menzies School of Health Research, Charles Darwin University, Darwin, Australia,

^7^Lao-Oxford-Mahosot Hospital-Wellcome Trust Research Unit, Vientiane, Lao PDR

†Equal contributors

[*Bipin@tropmedres.ac](mailto:*Bipin@tropmedres.ac)

**Journal**: Tropical Medicine and Health

| **Section/Topic** | **Item No** | **Checklist item** | **Reported on page No** |
| --- | --- | --- | --- |

# Semi-structured interview and focus group discussion guide for trial participants and community members

- Use this interview guide for interviews with health workers (

|  | | **SSI/FGD number:**  **Location:** | | | | | |
| --- | --- | --- | --- | --- | --- | --- | --- |
|  | | **SSI Note Takers Sheet** | | | | | |
|  | | **Note Takers initials:** | | | | | |
|  | | **Date: DD-MM-YYY** | | | | | |
|  | | **(town/settlement/neighborhood):** | | | | | |
|  | | **Socio-demographics** | | | | | |
| **Interviewer ID** | **Age** | | **Sex** | **Education (years)** | **Comments** | **Occupation** | **Respondent ID** |
|  |  | |  |  |  |  |  |
|  |  | |  |  |  |  |  |
|  |  | |  |  |  |  |  |
|  |  | |  |  |  |  |  |
|  |  | |  |  |  |  |  |
|  |  | |  |  |  |  |  |
|  |  | |  |  |  |  |  |
|  |  | |  |  |  |  |  |

- Avoid using closed questions and probe frequently
- **Obtain demographic data in the sheet below.**

1) Explain the study and ask for consent (memorise the verbal consent script as best you can), then record consent when you turn on microphone

*e.g “Thank you for seeing me today…are you happy to take part in this study?*

**Turn the recorder on**

*Ok, so I have turned the microphone on. I just wanted to ask again, are you happy to take part in this study by speaking with me today?*

2) Explore the themes below:

| **Topic** | **Suggested questions/probes** |
| --- | --- |
| **Tell me about your background** | - What do you do? - Which village are you from? How far is it from here? - What is your normal routine at home? |
| **About their past illnesses** | - What are the common illnesses that you suffer or your family members and the community? - What do you do when you suffer from illnesses? Where do you go for treatment? - What do you do when you have malaria? |
| **About malaria as their illnesses** | - Can you remember last time you had malaria? - How many times have you had malaria in the past? - Where did you get malaria from? - What were the symptoms of malaria? Can you describe? - How do you know you had malaria? - Do you know how many types of malaria are there? - What did you do? Where did you first go for treatment? - Did you receive treatment from a volunteer (probe: VMW/MMW)? - What types of medications have you received for malaria in the past? |
| **Impressions on MORU** | - Do you know the name of this clinic? - Have you heard about MORU/this clinic? - Do you remember visiting this clinic for treatment? Who referred you? - What do you remember about your last visit here? - How did you feel when you first came to MORU? - Are MORU staff friendly? - How did you come to MORU (by what means: motorbike)? - Was the travel to MORU difficult? And how long did it take? - Do you know the purpose of MORU? - How many times have you received services from MORU? - Do you know you participated in research/clinical trial? - Can you explain a bit about what you remember from the last visit? - Do you know what medicine were you provided (name of the medicine, colour, number)? - Did the medication cause side effects to you? - Is attending MORU for treatment expensive? - How many appointments did you have with MORU? - Did you have to pay any money to get treatment from MORU? - Did you find anything difficult to understand when you visited here? What were they? |
| **Information to participants** | - Can you explain to me how information was given to you when you visited the clinic here? - What did the MORU staff do when you arrived here? - Did you participate in the study in the clinic or you came for the treatment? Do you know the differences between two? Can you explain how are they different? - When you participated in MORU’s clinic, what was the reaction by your family member? What did they think of it? - What about blood test? What did you think about it? - How much blood did MORU staff take from you? How do you feel about it? - Were you scared of staying at MORU? (staying overnight for medication) - Did you receive information on how many days you should take the medicine given to you? - Do you have confidence in MORU’s treatment? - Do you think the information given to you were enough? - Did you fully understand the study you were participating? (probe: many respondents do not understand the entire concept of study, so adapt this question for a specific study) - Was it your decision to participate in the study? - Did your husband had opinion on your participation (did he influence the decision, if so, how?) - Did MORU staff motivated you to participate? - Did MORU staff forced you to participate? Or anyone else forced you to participate? |
| **Recommendations for information improvement** | - Would you like to recommend how we can improve the information about your participation in MORU clinic? (prompt: would visuals work better for you?) - How would you like us to visualize the information? |
| **CLOSING**  Summarise the key points from the interview | Do you have any questions or concerns you’d like to raise? Thank you. |
