# Supplementary material for: Community perceptions, trust, and experiences of malaria clinical research and participation in rural Cambodia
Source: Trop Med Health. 2026 Sep 8;54:193. doi: 10.1186/s41182-026-01069-4 (PMC13551720; doi:10.1186/s41182-026-01069-4)
Supplement: Supplementary file 3 [file 41182_2026_1069_MOESM3_ESM.docx]

# How is malaria research perceived in Cambodia? a qualitative study among study participants and community members

Bipin Adhikari^1,2†^, Lek Dysoley^3,4†^, Mom Ean^1†^, Hem Vattanak^1^, Ung Soviet^5^, Abhijit Mishra^1^, Sarah Cassidy-Seyoum^6^, Elke Wynberg^1^, Rupam Tripura^1,2^, Kamala Thriemer^6^, Cindy S Chu^2,7^, Nicholas PJ Day^1,2^, Lorenz von Seidlein^1,2^, Arjen M Dondorp^1,2^, Thomas J Peto^1,2^, Phaik Yeong Cheah^1,2^

^1^Mahidol-Oxford Tropical Medicine Research Unit, Faculty of Tropical Medicine, Mahidol University, Bangkok, Thailand

^2^Centre for Tropical Medicine and Global Health, Nuffield Department of Medicine, University of Oxford, Oxford, UK

^3^CNM National Centre for Parasitology, Entomology and Malaria Control, Phnom Penh, Cambodia

^4^School of Public Health, National Institute of Public Health, Phnom Penh, Cambodia.

^5^Provincial Health Department, Stung Treng, Cambodia

^6^Global and Tropical Health Division, Menzies School of Health Research, Charles Darwin University, Darwin, Australia,

^7^Lao-Oxford-Mahosot Hospital-Wellcome Trust Research Unit, Vientiane, Lao PDR

†Equal contributors

[*Bipin@tropmedres.ac](mailto:*Bipin@tropmedres.ac)

**Journal**: Tropical Medicine and Health

| Major theme | Sub-theme | Definition | Codes | Example Quote |
| --- | --- | --- | --- | --- |
| Knowledge and perceptions of malaria | Understanding of malaria transmission | Knowledge and beliefs about how malaria is transmitted | Mosquitoes, forest exposure, vectors, environmental risk | “It might be transmitted by mosquitoes or drinking unclean water which has mosquitoes’ eggs in it.” |
|  | Misconceptions about malaria | Incorrect or alternative explanations of malaria transmission | Drinking, water, sanitation, mosquito, eggs | “…drinking unclean water which has mosquitoes’ eggs in it.” |
|  | Lived experience of malaria | Personal experiences of repeated malaria infections and suffering | Recurrent illness, symptoms, burden of disease | “Countless, since I was 12 years old…” |
| Treatment-seeking experiences | Access barriers | Structural factors affecting healthcare access | Transport, distance, cost, road conditions | “I didn’t have a motorbike…” |
|  | Pluralistic treatment-seeking | Use of multiple healthcare providers and treatment sources | VMWs, military clinics, pharmacies, traditional care, self-treatment | Seeking care from multiple providers |
|  | Self-treatment and informal care | Initial attempts to manage illness outside formal healthcare | Home remedies, advice from family and friends and networks | Drinking beer and self-managing symptoms |
| Impressions of MORU research clinic | MORU as a healthcare provider | Perceptions of MORU as a clinic or treatment provider | Treatment, cure, healthcare delivery | “When I came to MORU, my symptoms will be recovered.” |
|  | Quality of care | Comparisons between MORU and routine healthcare services | Comfort, attentiveness, convenience, responsiveness | “I felt comfortable all of a sudden.” |
|  | Compassionate care | Perceptions of kindness, attentiveness, and communication by staff | Explanation, responsiveness, care, interactions | “He explained to us when we were confused…” |
|  | Community engagement and outreach | Perceptions of MORU’s activities beyond treatment | School visits, youth engagement, health education, posters, talks | “I saw them go to schools in the community…” |
| Understanding and experiences of research participation | Research-healthcare overlap | Perceptions that research and healthcare are intertwined | Research, perceived treatment, perceived health service | MORU perceived as helping poor people through treatment |
|  | Initial apprehension | Fear, anxiety, or uncertainty about participation | Fear, injections, unfamiliarity, cleanliness, quietness | “I was nervous too, to be honest.” |
|  | Altruistic motivations | Perceived broader purpose of research | Help communities, malaria elimination, poverty reduction | “MORU also wants to eliminate malaria…” |
| Follow-up and perceived efficacy of treatment | Follow-up as care | Viewing follow-up visits as evidence of good care | Regular, follow-up, phone call, ongoing support, compensation | “The doctor follows up long time…” |
|  | Understanding study purpose | Awareness of treatment comparisons and research objectives | Treatment comparisons, effectiveness, clear malaria, blood tests | “They want to know whether 7 days or 3 days is better.” |
|  | Perceived superiority of study treatment | Beliefs that study medicines are more effective | Better cure, side effects, lower recurrence | “The medicine at MORU doesn’t hurt…” |
| Trust and legitimacy | Trust in MORU | Confidence in MORU, staff, and services | Reliability, confidence, expectations of cure | Positive comparisons with other providers |
|  | Legitimacy of research activities | Perceptions of MORU as a credible and beneficial institution | Community benefit, authority, outreach, long-term presence, collaboration with health centre | MORU associated with helping communities |
| Recommendations for future engagement | Continuation of research and services | Desire for ongoing research and healthcare support | Recommendations, activities, sustain engagement | “Please continue to educate…” |
|  | Expansion of research scope | Calls for broader health research activities | Infectious and non-infectious diseases, dengue, typhoid, diabetes | “Not only malaria…” |
|  | Community health improvement | Research as a mechanism for improving population health | Health promotion, disease prevention, education | Calls for wider dissemination of knowledge |

## Analytic memos

### 1. Malaria as an embedded lived experience

It’s clear that participants' understanding of malaria was shaped not only by biomedical information they have received from formal sources but also by their own experience of living through it. Of course, not all of them had malaria, but they have seen it among their family members, friends and networks alike. That’s why malaria was described as a familiar and recurring condition, particularly among individuals who frequently entered forested areas. Those who have had malaria and who have acquired during their forest visits were the one to raise hands mostly during FGDs and share their experience. But they had different levels of knowledge related to malaria. Despite variations in knowledge regarding transmission, participants consistently recognised malaria as a significant burden in everyday life. This suggests that experiential knowledge may be as important as formal health education in shaping perceptions of malaria risk and healthcare utilisation.

### 2. Treatment-seeking as a pragmatic and sequential process

Treatment-seeking behaviour was complex but predictable too. Complex because their decision making is based on several social and household level factors, not to underestimate the critical role of whether they had cash or not. Predictable because, partly, my work in the past in Laos, Bangladesh, Cambodia and Nepal informed me more or less the pathways, how they navigate the treatment seeking behavior. Participants frequently moved between self-treatment, informal providers, village malaria workers, military clinics (only military personnels), health centres, and the MORU research clinic. These pathways appeared to be influenced by accessibility, cost, perceived effectiveness, and social recommendations. Rather than emerging from their literacy on health, such behaviours seem to suggest pragmatic decision-making.

### 3. Blurring of research and healthcare

A recurring yet a subtle undercurrent within these discussions often led to a theme that intersected on participants’ perception of research and clinical care. Across the interviews, there was a difficulty experienced by participants in distinguishing research activities from healthcare provision. MORU was often perceived as a malaria- specialised clinic, or a healthcare provider rather than solely a research institution. Research procedures such as informed consent, regular follow-up, and clinical monitoring were frequently interpreted as a form of care. This overlap between research and care appears central to understanding participant experiences and may reflect elements of therapeutic misconception which is also a core spinal finding in this study.

### 4. Trust as a product of care and engagement

If that’s how participants conflated between research and care, my interpretation of trust has to be cautious, and contested. Trust emerged as a cross-cutting theme underpinning participants' perceptions of MORU. Trust appeared to be built through multiple mechanisms, including repeated interactions with staff, perceived treatment efficacy, attentive care, follow-up visits, and community engagement activities. Trust therefore appeared to extend beyond clinical outcomes and was closely linked to participants' experiences of respect, communication, and responsiveness.

### 5. Follow-up as both research procedure and care practice

I was fascinated by how follow-up was perceived by participants. Participants often interpreted follow-up visits not as research requirements but as evidence of professional commitment and concern for their wellbeing. Follow-up therefore appeared to serve dual functions: generating research data while simultaneously reinforcing perceptions of care quality and trust. This dual interpretation may have contributed to positive perceptions of both the research clinic and participation in research.

### 6. Research participation as community benefit

Participants frequently framed research participation in collective rather than individual terms, although I am not sure, how much participants have articulated this out of social desirability biases. The fact that some of them know that research has a wider benefit to the society. Research was often viewed as contributing to malaria elimination, helping poor communities, improving local health services, and increasing access to healthcare. These findings suggest that participation was partly motivated by perceived community benefit and broader social goals rather than solely by individual treatment outcomes.

### 7. Expectations for continued engagement

Some of the participants are the parents of youth advisory group on health and research engagement (YAGHRE) members and I can clearly understand how much they appreciate such a program and thus I do use caution in interpreting this highly positive recommendation. Recommendations for future work reflected strong community expectations that MORU's role should extend beyond malaria research. Participants expressed interest in continued health education, broader disease-focused research, and ongoing access to high-quality healthcare services. These expectations suggest that long-term engagement may shape how communities understand the role and responsibilities of research organisations operating in resource-constrained settings.
